# Simulation of gas sensing mechanism of porous metal oxide semiconductor sensor based on finite element analysis

**DOI:** 10.1038/s41598-021-96591-2

**Published:** 2021-08-25

**Authors:** Songlin Li, Min Zhang, Hai Wang

**Affiliations:** School of Aerospace Science and Technology, Xidian University, 2 Taibai South Road, Xi’an, 710000 China

**Keywords:** Chemical physics, Techniques and instrumentation

## Abstract

In recent years, finite element analysis is increasingly adopted to simulate the mechanism of metal oxide semiconductor (MOS) resistive gas sensors. In this article, the chemical reaction engineering module in the COMSOL Multiphysics tool is used to describe the dynamic equilibrium process of oxygen ions in the sensor. The boundary conditions of temperature transfer, conductivity model, and mass transfer are applied to simulate the convection, diffusion, and penetration processes. The response of the sensor at different temperatures (445 K–521 K) and different target gas concentrations (1–500 ppm) is simulated. In this paper, the dynamic model of oxygen ions is used creatively as a bridge between gas concentration and sensor response instead of the traditional direct parameter fitting method. The simulated result of the surface oxygen ion control and permeability control model of the MOS gas sensor shows a good agreement with the real sensor. For explaining the principle of metal oxide semiconductor gas sensors simulations has been performed on COMSOL Multiphysics software. The proposed method in this paper is based on the underlying transfer logic of the sensor signal, it is expected to predict the sensor signal and assist the sensor design.

## Introduction

Since the first report of MOS gas sensor by Tetsuro Seiyama in 1962^1^, both the preparation methods^2,3^ and the sensing mechanism^4,5^ of MOS gas sensor have been increasingly studied. It has been reported^6^ that the interconnected micropores and abundant active sites of PdO-doped three-dimensional ZnFe2O4 nanospheres equip the sensor with faster response and selectivity to target gas. Reference^7^ found that SnO_2_ nanospheres with a three-dimensional hierarchical structure have an ultra-high response to ethanol due to the abundant active sites^8^ on the sensor surface. However, the preparation of resistive MOS gas sensors faces the problem of low efficiency and high complexity^9^. Research on the time-response characteristics of sensors using the finite element model method (FEM) can effectively help solve these problems^10,11^. At present, there are a lot of finite element studies for SAW^12,13^ and BAR^14^, but there are fewer finite element methods based on chemical reaction engineering for MOS.

Accurate simulation of MOS gas sensors is essential for device design. Reference^15,16^ simulate the impact of sensor microstructure on the response but overlook the chemical reaction occurring on the sensor’s surface. Reference^17^ adopts a circuit structure to equivalently replace the physical and chemical reactions of the sensor surface, which simplifies the sensor signal conduction model and, the conversion of the sensor between chemical signals and physical signals. Reference^18–21^ fits the response curve to a related function to simulate the sensor. The work unifies the influencing factors of the sensor but lacks the explanation of the material and signal transmission of the sensor.

In this article, the finite element analysis instrument of COMSOL was adopted to simulate and analyze the time response characteristics of the MOS gas sensor. The chemical reaction engineering module was applied to simulate the surface oxygen ion dynamic adjustment model^22^. The boundary conditions of temperature transfer, conductivity model, and mass transfer were utilized to simulate the convection and diffusion process. The homogenization model was introduced to simulate the permeation effect on the porous surface. This simulation method is closer to the underlying signal transmission logic. The model’s time response curve of the sensor and Liu’s experimental results^23^ has reached an amazing consistency. The effective verification of the oxygen ion control model and the permeation model is expected to provide useful guidance for the design and manufacture of MOS gas sensors^24^.

## Sensing principle

Exposure of target gas concentration to the sensor changes the resistance of sensing layer. As its surface is converted by oxygen ions, the sensor resistance returns to the initial value. The response of MOS sensor is affected by a series of factors, such as the number of surface oxygen ions, permeability^25^, sensor film thickness, temperature, and gas concentration. Reference^26,27^ shows that the oxygen ion concentration on the sensor surface changes with different annealing temperature and noble metal doping, and the resistance value of the sensor in the air environment. Reference^23,28^ shows that the porous interconnected sensor surface increases the gas permeability, adsorption, and desorption rate, which affects the sensor's response time and recovery time. Reference^29,30^ shows that the sensor film thickness is related to the sensor's performance. Temperature and concentration have an impact on the rate of a chemical reaction. Reference^31,32^ shows that temperature and concentration are also related to permeability. It is widely accepted that the response of the sensor is directly related to the surface reaction^33^ and surface permeability^34^. When the sensor stays in the air environment, oxygen on its surface is adsorbed to form surface oxygen ions in large quantities (${\mathrm{O}}_{2}+2{\mathrm{e}}^{-}=2{\mathrm{O}}^{-}$). Taking a N-type semiconductor gas sensor as an example, the active sites capture oxygen to generate surface oxygen ions, which consumes the carrier electrons inside the sensor. Currently, the sensor maintains a high resistance. When the sensor contacts the target gas environment, the target gas penetrates the sensor surface and react with the adsorbed oxygen ions on the surface, and the reaction formula is as follows:

1 $${Gas}_{Target}+{O}^{-}={Gas}_{Product}+{e}^{-}$$

**Figure 1:**
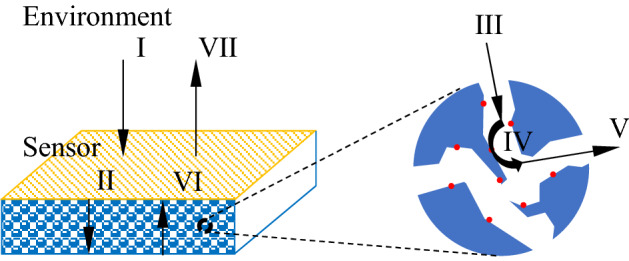
Mass transfer model of the sensor surface reaction. I: The measured substance transferred from the environment to the sensor surface; II: The measured substance diffuses from the through-hole of the sensor to the internal surface of the sensor; III: The measured substance Adsorb on the sensor surface; IV: The measured substance reacts with the surface substance; V: The reacted substance desorbed from the sensor surface; VI: The reaction product diffuses from the through-hole of the sensor to the sensor surface; VII: The product moves from the sensor surface to the environment.

In this way, the surface oxygen ions are consumed in a large amount, with many active sites released and, electrons returning to the sensor, causing an increase in the carrier concentration and thus a low sensor resistance. Different concentrations of target gas cause the two chemical reactions to produce different dynamic balances. The sensor sensing process is very complex and involves many physical and chemical processes. In particular, it is very important that the dissociative gas interaction with the sensitive surface. The simulation model of this paper simplifies the sensor sensing process. Given the complexity of modeling, it is neglected that the reaction product reacts again with the surface in this paper. The reaction is divided into seven steps^35^:

From Fig. 1 we can see that steps I and VII take place at the macro level, while steps II and VI take place in porous media, and steps III–V take place on the inner surface. Therefore, the semiconductor sensor principle can be divided into the physical process and the chemical process. The physical process is the diffusion of gas into the porous interconnected metal oxide sensor surface through laminar flow, turbulent flow, and porous media; the chemical process is that the measured gas diffuses into the sensor, and then the surface of the sensor reacts with oxygen ions, which in turn causes the surface oxygen ion concentration to change.

## Model design

Many factors affect the response of MOS sensors, but different physical and chemical processes can be comprehensively considered through finite element analysis to simulate the sensor's sensing mechanism and predict the sensor signal. This paper creatively verified the oxygen ion model of the resistive MOS sensor through the finite element simulation method and added a homogenization model on this basis to introduce the gas diffusion process into the model.

Based on the sensing mechanism of the sensor, COMSOL Multiphysics is used to model and analyze the physical and chemical reactions of the sensor. Here, the chemical reaction engineering module of the sensor is modeled first, and then the physical processes are added to model the sensor.

## Design of chemical reaction engineering module

According to the surface ion reaction of step IV and formula (1), we define here that both reactions are irreversible, and the chemical reaction equation analyzes according to the sensing mechanism and Liu’s experimental.

2 $${CH}_{3}CO{CH}_{3}+8{O}^{-}=3{H}_{2}O+3C{O}_{2}+8{e}^{-}$$

3 $$4{O}_{2}+8{e}^{-}=8{O}^{-}$$

Here the active site fraction occupied by molecular oxygen adsorption into oxygen ions is set to *θ* ($0<\theta <1$). Langmuir derived that the molecular desorption rate is proportional to θ. The adsorption rate of oxygen into oxygen ions is proportional to ($1-\theta$), where the consumption and generation rate of oxygen ions can set as (4, 5).

4 $${r}_{con}={k}_{f1}\times {P}_{a}\times \theta$$

5 $${r}_{gen}={k}_{f2}\times {P}_{a}\times \left(1-\theta \right)$$

Among them, ${k}_{f1}$ and ${k}_{f2}$ are constant at a specific temperature, and ${P}_{a}$ is the partial pressure of the gas. Wolkenstein^36^ postulated that the number of adsorption centers on the surface is a constant characterizing the surface and depending on its history. To set up transport and reaction equations in terms of *c* (sensor gas concentration of acetone or oxygen in mol/m^3^), ${c}_{S}$ (surface concentration of oxygen ions), ${T}_{S}$ (the total surface concentration of active site), make the following substitutions:

6 $$\theta ={c}_{S}/{T}_{S}$$

7 $${P}_{a}=cRT$$

where *R* is the gas constant, and *T* is the temperature. Since the oxygen concentration in the air is constant, ${r}_{con}$ (consumed surface oxygen ions) and ${r}_{gen}$(generate surface oxygen ions) are derived as:

8 $${r}_{con}={k}_{f1}\times {c}_{T}\times R\times T\times {c}_{S}/{T}_{S}$$

9 $${r}_{gen}={k}_{f2}\times {c}_{O}\times R\times T\times \left({T}_{S}-{c}_{S}\right)/{T}_{S}$$

**Figure 2:**
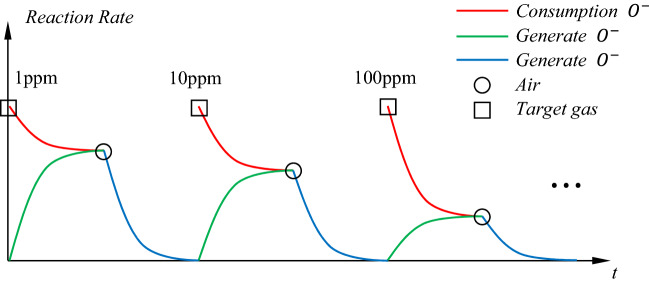
A diagram of the rate of chemical reactions at different acetone concentrations. The squares and circles represent the introduction of target gas and air into the test environment, respectively. The red line represents the rate of consumption oxygen ions. The blue and green lines indicate the rate of generation oxygen ion in the presence/absence of competing reactions.

Among them,${c}_{T}$ and ${c}_{O}$ are the concentrations of acetone and oxygen in the sensor, respectively. Here, the consumption and generation rates of oxygen ions are in dynamic equilibrium. The schematic diagram of the reaction rate of consumption and generation of oxygen ions is shown in Fig. 2. The red line represents the rate of consumption oxygen ions. The blue and green lines indicate the rate of generation oxygen ion in the presence/absence of competing reactions. When the acetone concentration changes, the equilibrium position of the two competing reactions will change accordingly. In other words, the percentage of oxygen ions occupying the active area of the sensor changes with the acetone concentration. Here the difference function $F({c}_{T})$ is used to correct the process.

10 $${r}_{con}={k}_{f1}\times {c}_{T}\times R\times T\times ({c}_{S}-F({c}_{T})\times {T}_{S})/{T}_{S}$$

11 $${r}_{gen}={k}_{f2}\times {c}_{O}\times R\times T\times \left(k\times {T}_{S}-{c}_{S}\right)/{T}_{S}$$

The response time of the sensor time resistance characteristic is closely related to the recovery time and the chemical reaction rate. Then the value of ${k}_{f1}$ and ${k}_{f2}$ is corrected by extracting the response time recovery time of the experimental data.

The molecular molar mass in Table 1 gets from the periodic table of chemical elements. The oxygen concentration is the oxygen concentration in the air. The active site and target gas concentration are gained from the simulation content. Next, the physical model is built based on the chemical reaction engineering module.

**Table 1:** Sensing principle parameters.

Parameter	Description	Value	Unit
M_O2	Oxygen molar mass	0.032	kg/mol
M_O	Oxygen ion molar mass	0.016	kg/mol
M_N2	Nitrogen molar mass	0.028	kg/mol
M_H2O	Water molar mass	0.018	kg/mol
M_CO2	Carbon dioxide molar mass	0.044	kg/mol
M_target	Target gas molar mass	0.048	kg/mol
C_ts	Active site concentration	50	mol/m^2^
C_O2	Oxygen concentration	9.6875	mol/m^3^
C_O0surf	Surface oxygen ion concentration	C_ts × 0.9	mol/m^2^
C_target	Target gas concentration	PPM × 2.577e−5	mol/m^3^
PPM	Parts per million	1–500	/

### Design of environment physical model

**Figure 3:**
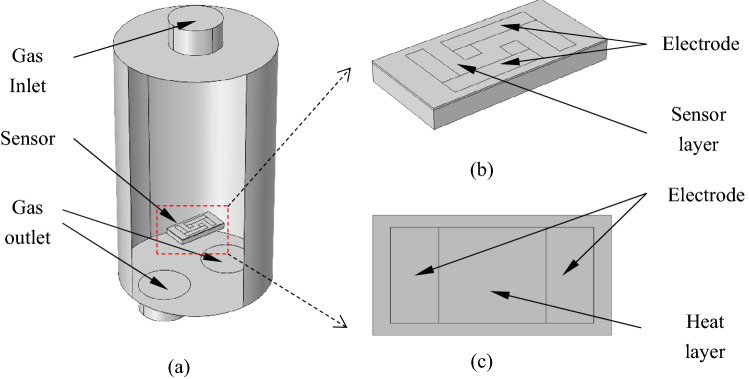
(**a**) Sensor and gas cavity model. (**b**) Electrode and sensor layer model on the front of the sensor. (**c**) Heating electrode model on the back of the sensor.

The physical environment model is generated based on the chemical reaction engineering model. Firstly, built the sensor environment model. The specific model and parameters are shown in Fig. 3 and Table 2. As shown in Fig. 3, there is one inlet on the top and two outlets on the bottom, with the sensing element arranges in the lower part of the middle. The top of the sensor is the sensing part and the resistance measuring electrode, the middle is the substrate, and the bottom is the heating part and the heating electrode. The Mesh View used for the simulation of the structures is shown in Figs. S1 and S2.

**Table 2:** Model geometric parameters.

Parameter	Description	Value	Unit
cyl_H	Gas chamber height	10	mm
cyl_R	Gas chamber radius	3	mm
in_R	Gas input channel radius	1	mm
tube_H	Gas input/output channel height	1	mm
out_R	Gas output channel radius	1	mm
chip_L	Length of the sensor substrate	1	mm
chip_W	Width of the sensor substrate	2	mm
chip_H	Height of sensor substrate	0.1	mm
Front_H	Sensing layer thickness	0.01	mm

The shape parameters of the gas chamber and the active sensor layer were obtained according to reference^9,23^.

Considering the three processes, i.e. heat transfer, mass transfer, and reaction, we add a chemical module, a heat transfer module, a laminar flow module for step I and VII, a dilute substance transfer module for step II and VI, a current module, and a Multiphysics module for the responsibilities involved in the sensor. The chemical reaction engineering module simulates the reaction on the sensor surface. The heat transfer module simulates the heat transfer in sensor and air. The dilute substance transfer module simulates the mass transfer of porous media inside the sensor. The laminar flow module simulates the gas flow in the gas cavity. The current module simulates the resistance value of the sensor surface. The Multiphysics module is responsible for coordinating each module.

**Figure 4:**
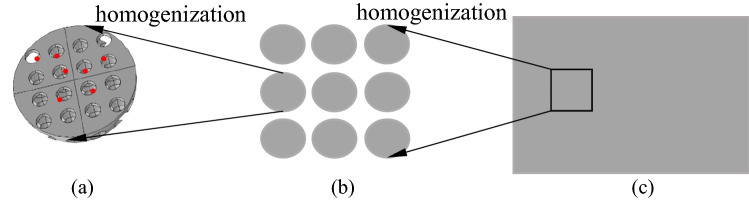
(**a**) The microstructure particles of the sensor material and the reaction site attached on it. (**b**) The accumulation of MOS nanoparticles after homogenization. (**c**) Further homogenization of the material macroscopic layer.

For mass transfer and chemical reactions on the sensor surface, because of the large number of nanoscale porous microspheres with three-dimensional (3D) interconnected holes on the sensor’s surface, it is impossible to model the specific mechanism one by one. In this case, for steps III and V, we can model the porous structure through homogenization. Essentially, this means that we treat porous particles as uniform plates containing fluid and catalyst. Instead, porosity is adopted to model the fluid, and its useful transport characteristics depend on the porosity and tortuosity of the particles^31,32^. The homogenization model shows in Fig. 4. The exposure of the target gas is simulated by coupling the gas chamber domain shows in Fig. 3a with the porous media domain use of homogenized models.

Due to the simultaneous use of chemical reaction engineering and the physical environment model to couple mass transfer, the simulation convergence speed was very long, so the model is divided into acetone environment and air environment. Take the calculation result of the acetone environment as the initial value of the air environment. Then the simulation time for transient studies will be reduced from weeks to days, using Intel(R) Xeon(R) Gold 5218 CPU and at least 16 GB of memory.

The current voltage module is used to simulate time resistance characteristics. Studies^37^ have shown that the surface properties of a material have a strong correlation with the resistance of the material, here we define resistivity as a function of the ratio of oxygen ions. Then the relation between target gas concentration and change in sensor resistivity is established by the concentration of oxygen ions on the surface.

## Results and discussion

**Figure 5:**
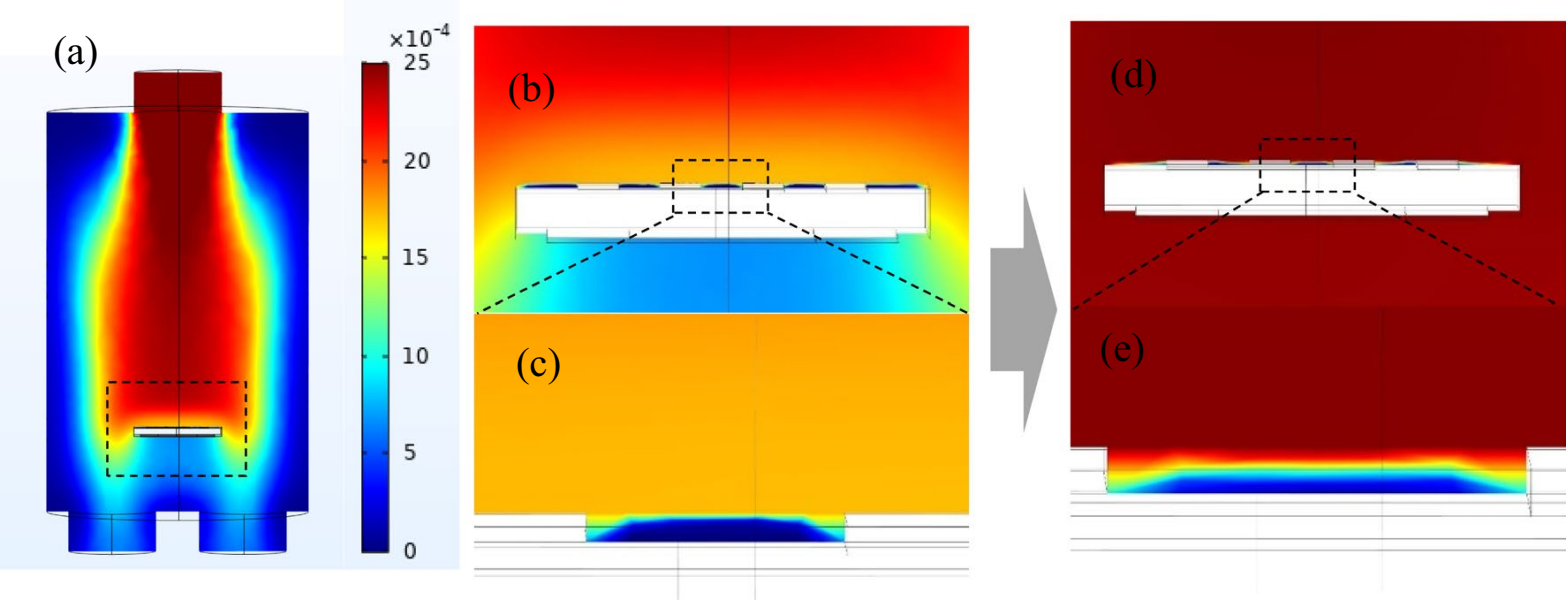
(**a**) Transient concentration distribution of acetone gas. (**b**) The acetone concentration distribution in the early time. (**c**) Partial enlargement of (**b**). (**d**) The acetone concentration distribution at the next moment of (**b**). (**e**) Partial enlargement of (**d**).

To test this method, we model the sensor and compare the simulation data with the experimental data^23^. In this work, they produced α-Fe2O3 with a mesoporous structure as a sensitive material to detect acetone gas. Here N-type semiconductor porous metal oxide is used as the sensing part to evaluate the performance of the reducing acetone gas. The transient concentration distribution of acetone gas introduced into the sensor chamber is shown in Fig. 5. Rainbow bars indicate the concentration of acetone. Figure 5a is the concentration distribution of acetone gas in the gas cavity. Figure 5b is the local acetone concentration distribution of the sensor substrate. Figure 5c is the acetone concentration distribution in the sensor layer currently. Figure 5d and e is the acetone concentration distribution at the next moment. It can be seen from Fig. 5 that the propagation speed of acetone gas in the gas chamber is much higher than that in the porous medium of the sensor. The homogenization model proposed in this paper can be used to simulate the material transfer characteristics of the sensor sensing medium. The heat transfer, dilute substance transfer, and laminar flow module can simulate the material transfer in the gas chamber.

**Figure 6:**
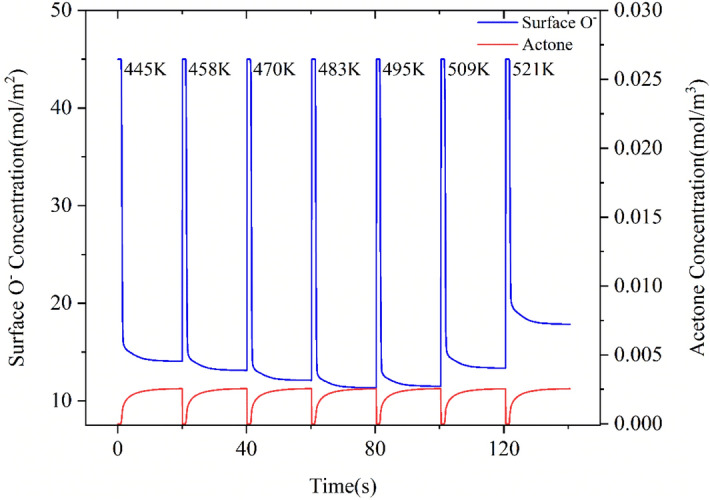
Transient distribution of surface oxygen ion concentration on the sensor surface at different temperatures, the blue line is the acetone concentration distribution inside the sensor under the action of diffusion and convection.

With the propagation of acetone, the number of surface oxygen ions in the porous medium decreases rapidly. The change of oxygen ion concentration on the sensor surface at different temperatures is shown in Fig. 6, where the red line shows the change curve of acetone concentration in the porous medium. It can be seen from Fig. 6 that with the introduction of acetone, surface oxygen ions are rapidly consumed. Moreover, the surface oxygen ion concentration changes with the temperature and concentration of acetone.

**Figure 7:**
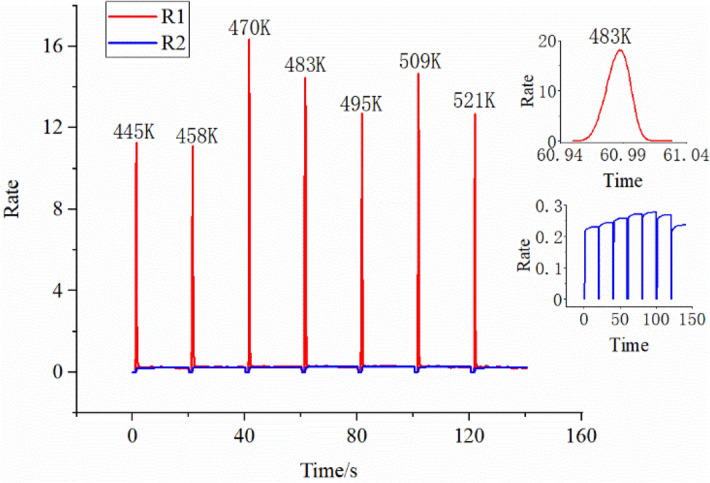
Rate of chemical reaction that generates (R1) and consumes (R2) oxygen ions. The red inset curve is a zoomed-in view of the chemical reaction rate of oxygen ion generation at a temperature of 483 K. The blue inset curve is a zoomed-in view of the chemical reaction rate of oxygen ion consumption at different temperatures.

The change curve of the chemical reaction rate between consumption and production of oxygen ions at different temperatures is shown in Fig. 7. The red curve R1 represents the chemical reaction rate of oxygen generates electrons to produce oxygen ions. The blue curve R2 represents the chemical reaction rate of acetone reacting with surface oxygen ions to consume oxygen ions. The blue inset curve in Fig. 7 is an enlarged view of dynamic changes. From the blue inset curve in Fig. 7, the dynamic balance of the chemical reaction rate of consumption and production of oxygen ions changes with temperature. The red inset curve in Fig. 7 is a zoomed-in view of the chemical reaction rate of oxygen ion generation at a temperature of 483 K. The maximum chemical reaction rate of oxygen ions generated at different temperatures varies irregularly. This is because the rate of chemical reaction to generate oxygen ions is not only related to the ratio of chemical reaction sites but also to the concentration of acetone that penetrates the sensing medium. Therefore, it can be seen from the red inset curve that the chemical reaction rate of oxygen ion generation firstly increases with the increase of acetone concentration and then decreases due to the decrease of chemical reaction sites.

**Figure 8:**
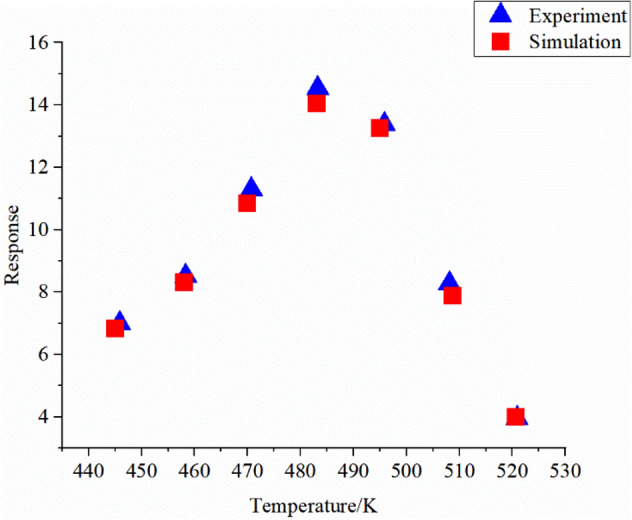
Response of the experiment and simulation results at different temperature.

The temperature response of the sensor and the simulation result is shown in Fig. 8.

The triangle scatters represent is the experimental data while the square ones are the simulation data. Here, the response of 100 ppm acetone at different temperatures is selected. In the Fig. 8, the response initially increases and then decreases as the temperature increases. In the low temperature region, as the temperature increases, more reaction sites will participate in the reaction. When the temperature is too high, the reaction sites are destroyed, and the response will decrease rapidly. Here we use formula (10) to calculate the fitting index between the experimental data and the simulated data.

12 $${R}_{new}=1-{\left(\frac{\sum {(y-{y}^{*})}^{2}}{\sum {y}^{2}}\right)}^\frac{1}{2}$$

where *y* is the experimental data, ${y}^{*}$ is the simulated data, ${R}_{new}$ is the fitting index. The fitting index between the experimental data and the simulated data reaches 0.97, indicating that the simulation is very accurate.

**Figure 9:**
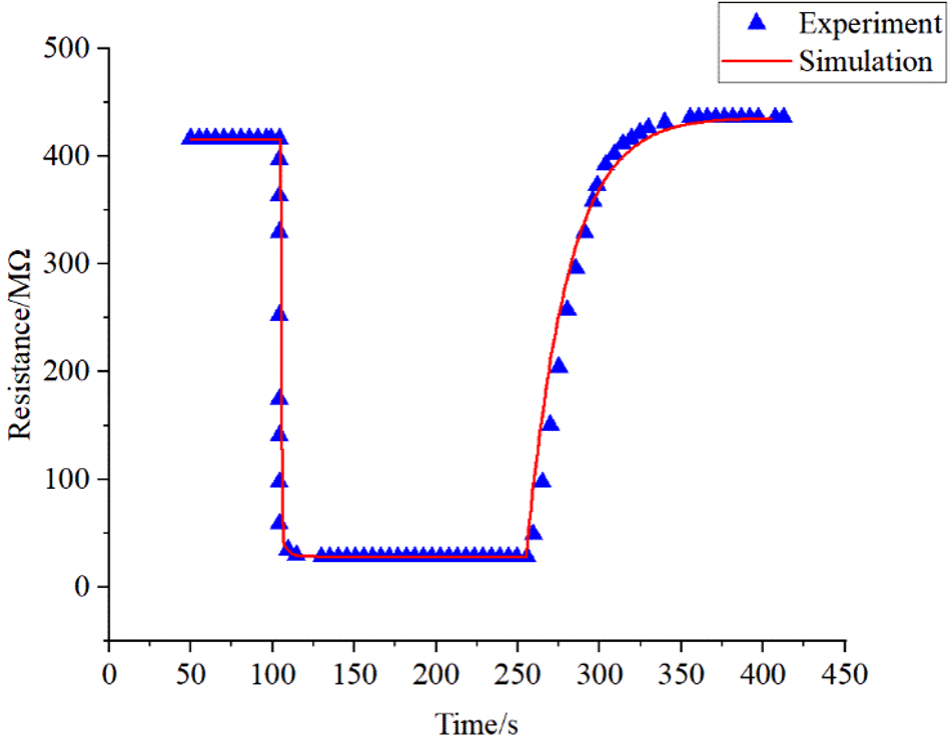
Comparison of simulation and experimental time resistance curves of 100 ppm acetone at 483 K.

Compare with the experimental results, here we first select the heating temperature when the response is maximum as the next simulated temperature. The comparison between the simulation and experiment results when the acetone concentration is 100 ppm at 210 °C shows in Fig. 9.

As shown in Fig. 9, the triangle scatters are the experimental data, and the line is the simulation data. 100 ppm of acetone is passed in at 105 s and stops at 255 s. As described in Sect. 3.1, in order to speed up the convergence of the model, the time resistance characteristics are divided into acetone environment and air environment, wherein the calculation results of acetone environment as the initial value of the air environment. Due to measurement errors and long-term sensor stability, a baseline after exposure to target gas will exceed it before exposure. Here we correct the parameters in this model according to the differences in experimental data. Before the acetone passed into the gas chamber, the air contains much oxygen, and the surface of the sensor contains a lot of active sites. The total number of active sites and the number of oxygen ions occupy a dynamic balance. The number of oxygen ions occupying the active site no longer changes, and the resistance value is stable. After passing acetone gas into the chamber, oxygen ions on the surface of the sensor are consumed rapidly, and the resistance of the sensor decreases rapidly. When the consumption and generation rates of oxygen ions are balanced, the number of oxygen ions on the sensor surface and the resulting resistance value will reach a stable state. The fitting index calculated from formula 10 is 0.99, indicating that the model can simulate the sensor well. It can also be seen in Fig. 9 that the experimental data and the simulated data are consistent.

**Figure 10:**
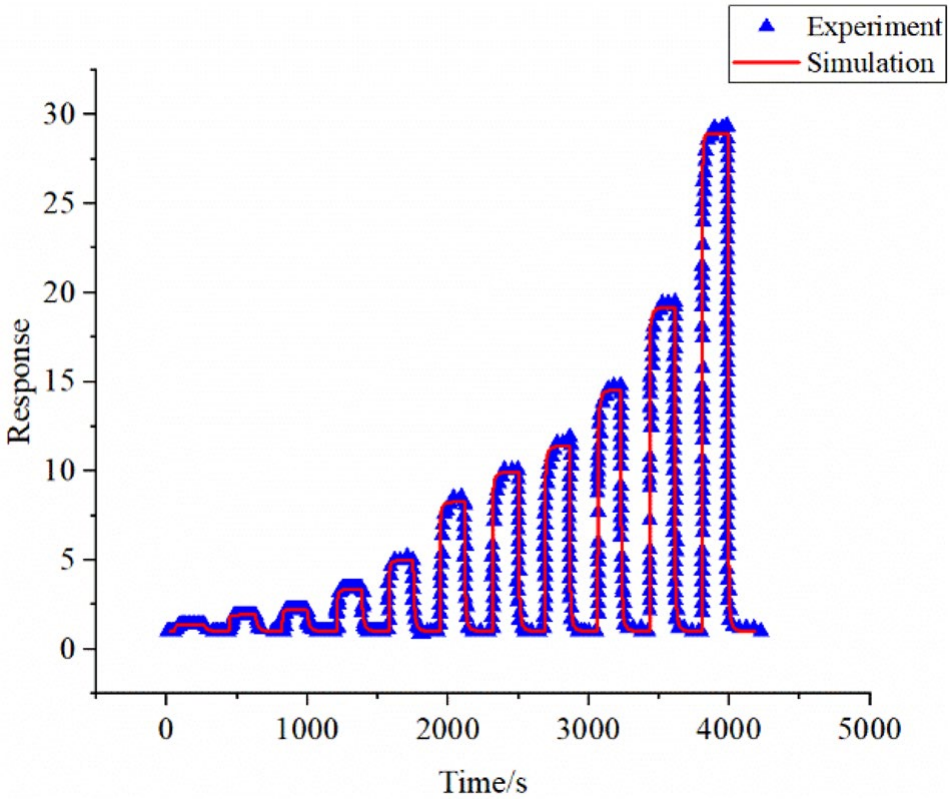
Comparison of simulation and experimental response curves of 1–500 ppm acetone at 483 K.

When the concentration of acetone changes, the equilibrium level also changes. The simulation results and experimental data of 1–500 ppm are shown in Fig. 10, the simulation results indicate that the saturation effect emerges when the gas concentration is higher than 500 ppm. Limited by experimental data, the performance of our model under the saturation effect is not evaluated. Based on the surface oxygen ion control and permeability control model in this work, we believe that the situation of saturation effect is also applicable.

In the Fig. 10, the point where the curve rises from 1 is the acetone entry point, and the point where the curve drops is the time when the acetone stopped. When acetone gas of different concentrations is introduced, the speed of diffusion into the sensor is different, which leads to the change of the oxygen ion consumption rate on the sensor surface. Moreover, the introduction of different concentrations of acetone gas causes the change of the acetone concentration inside the sensor when the diffusion is stable. The concentration of acetone gas inside the sensor changes the balance of ion consumption and generation on the sensor surface. Different concentrations of acetone gas change the response of the sensor by affecting the diffusion and surface reaction. This model simulates surface reactions through chemical reactions and simulates diffusion effects through a uniform model. The fitting index value between the simulation and experimental data is 0.98. From the calculation results, we can get an accurate prediction of the response of the porous sensor under different concentrations of acetone. Figure 10 can also indicate that the method of combining the active site and diffusion theory proposed in this paper to model the sensor behavior can better explain the experiment. Next, through the continuous iteration of experimental data and simulation, the simulation model can be optimized, and the experiment can also be guided and designed greater.

## Conclusion

This work explains the use of the concept of active sites to introduce the chemical reaction module combined with the convection, diffusion, and penetration model to simulate the characteristics of the sensor using finite element methods. In this work, the time-resistant characteristics of MOS gas sensors are modeled and analyzed by using finite element methods in combination with chemical reaction engineering and physical transfer models with COMSOL for the first time. The response of the sensor simulation model and the fitting degree of the experimental data at different temperatures (445 K–521 K) and different target gas concentrations (1–500 ppm) reached 0.95. It shows that the model proposed in this paper can simulate the characteristics of the sensor. With the continuous iteration of simulation and experiment, this module is expected to guide the development of experimental sensors and significantly reduce the difficulty of sensor development. In future work, we will improve the model to simulate the microscopic mass transfer process of the sensor in the mass transfer process, and the dissociative gas interaction with the sensitive surface for more accurate analysis and design of the sensor behavior.

## Supplementary Information

Supplementary Information.
