# Supplementary material for: Simulation of gas sensing mechanism of porous metal oxide semiconductor sensor based on finite element analysis
Source: Sci Rep. 2021 Aug 25;11:17158. doi: 10.1038/s41598-021-96591-2 (PMC8387441; doi:10.1038/s41598-021-96591-2)
Supplement: Supplementary file 1 — Supplementary Information. [file 41598_2021_96591_MOESM1_ESM.pdf]

Supporting Information for

Simulation of gas sensing mechanism of porous metal oxide semiconductor sensor based on finite element analysis

Li Songlin[1], Zhang Min[1], Wang Hai[1]*

[1]School of Aerospace Science and Technology, Xidian University, 2 Taibai South Road, Xi'an, 710000, China

*Author to whom correspondence should be addressed: wanghai@mail.xidian.edu.cn

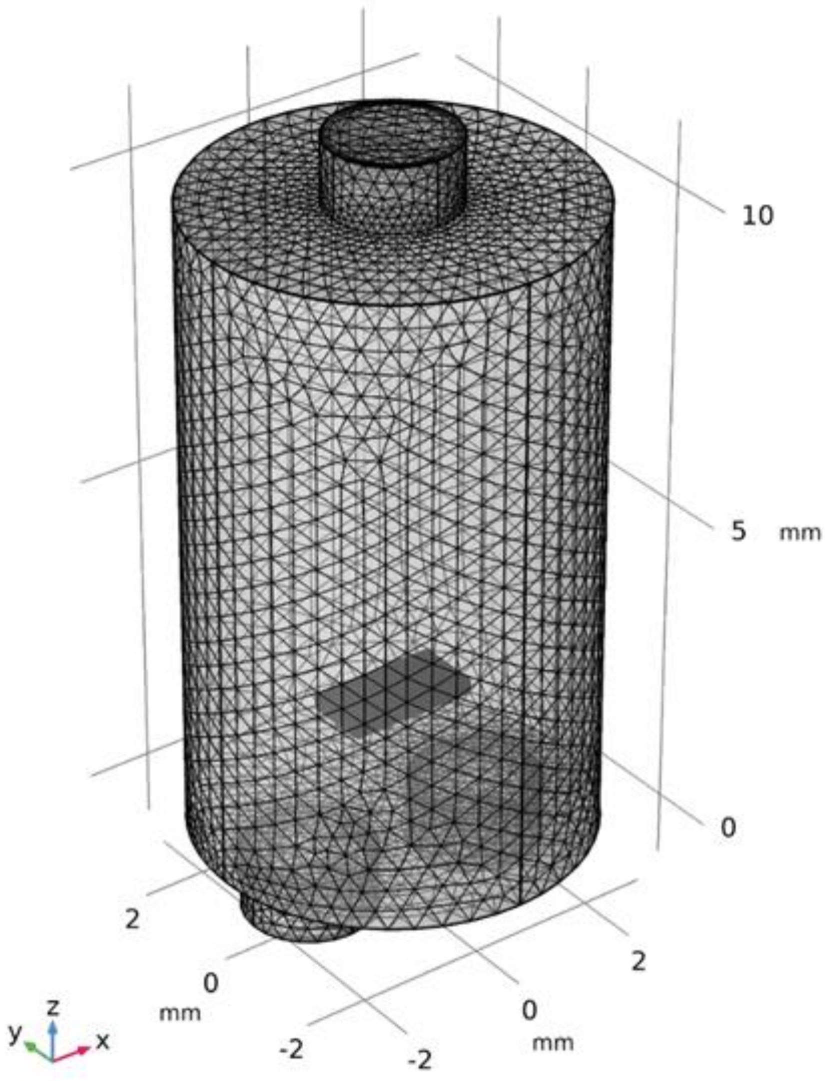

Fig.S1. The Mesh View used for the simulation of the gas chamber.

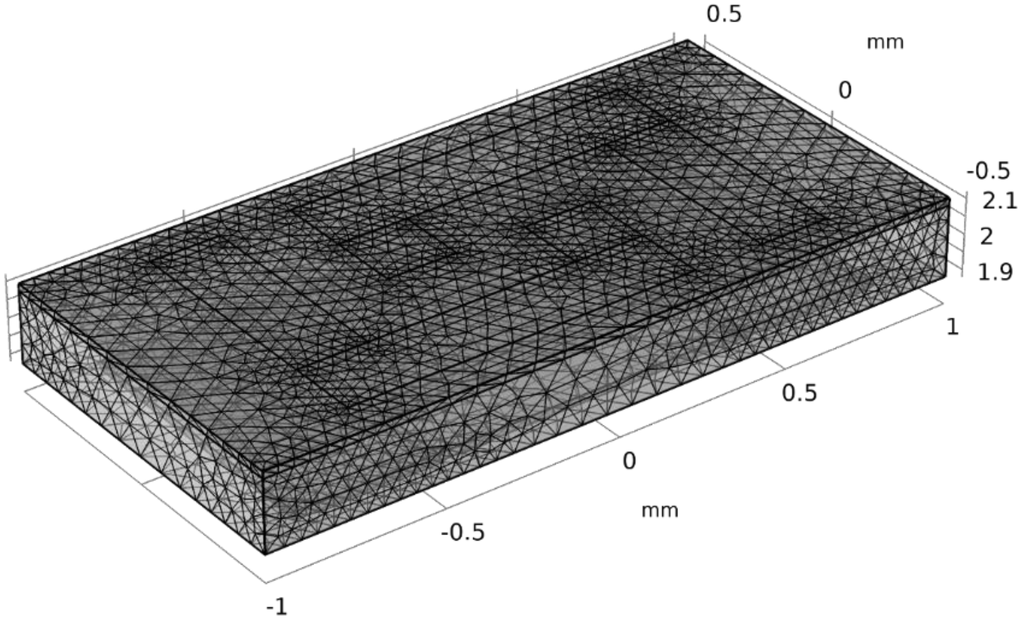

Fig.S2. The Mesh View used for the simulation of the gas sensor.
